# Clinical Outcomes Before and After Prucalopride Treatment: An Observational Study in Patients With Chronic Idiopathic Constipation in the United States

**DOI:** 10.14309/ctg.0000000000000687

**Published:** 2024-02-15

**Authors:** Anthony Lembo, Brooks D. Cash, Mei Lu, Emi Terasawa, Brian Terreri, Shawn Du, Rajeev Ayyagari, Paul Feuerstadt, Baharak Moshiree, Ben Westermeyer, Selina Pi, Mena Boules

**Affiliations:** 1 Digestive Disease Institute, Cleveland Clinic, Cleveland, Ohio, USA;; 2 University of Texas Health Science Center at Houston, Houston, Texas, USA;; 3 Takeda Pharmaceuticals USA, Inc., Lexington, Massachusetts, USA;; 4 Analysis Group, Inc., New York, New York, USA;; 5 Analysis Group, Inc., Boston, Massachusetts, USA;; 6 PACT Gastroenterology Center, Hamden, Connecticut, USA;; 7 Yale School of Medicine, Yale University, New Haven, Connecticut, USA;; 8 Atrium Health Wake Forest University School of Medicine, Charlotte, North Carolina, USA.

**Keywords:** chronic idiopathic constipation, prucalopride, real-world, symptoms, complications

## Abstract

**INTRODUCTION:**

This real-world US-based claims study compared constipation-related symptoms and complications 6 months before and after prucalopride initiation in adults with chronic idiopathic constipation (CIC).

**METHODS:**

This observational, retrospective cohort analysis used the IBM MarketScan Commercial Claims and Encounters Database and the Medicare Supplemental Database (January 2015–June 2020). Prucalopride-treated patients (≥18 years old) who had ≥1 constipation-related *International Classification of Diseases, Tenth Revision, Clinical Modification* (*ICD-10-CM*) diagnosis code during the baseline or study period were included. The proportions of patients with constipation-related symptoms (abdominal pain, abdominal distension [gaseous], incomplete defecation, and nausea) and constipation-related complications (anal fissure and fistula, intestinal obstruction, rectal prolapse, hemorrhoids, perianal venous thrombosis, perianal/perirectal abscess, and rectal bleeding) were examined. Constipation-related symptoms and complications were identified using *ICD-10-CM*, *ICD-10*-*Procedure Coding System*, or Current Procedural Terminology codes. Data were stratified by age (overall, 18–64 years, and ≥65 years).

**RESULTS:**

This study included 690 patients: The mean (SD) patient age was 48.0 (14.7) years, and 87.5% were women. The proportions of patients overall with constipation-related symptoms decreased 6 months after prucalopride initiation (abdominal pain [50.4% vs 33.3%, *P* < 0.001]; abdominal distension [gaseous] [23.9% vs 13.3%, *P* < 0.001]; and nausea [22.6% vs 17.7%, *P* < 0.01]; no improvements observed for incomplete defecation). Similarly, the proportions of patients overall with constipation-related complications decreased 6 months after prucalopride initiation (intestinal obstruction [4.9% vs 2.0%, *P* < 0.001]; hemorrhoids [10.7% vs 7.0%, *P* < 0.05]; and rectal bleeding [4.1% vs 1.7%, *P* < 0.05]).

**DISCUSSION:**

This study suggests that prucalopride may be associated with improved constipation-related symptoms and complications 6 months after treatment initiation.

## Graphical abstract

**Figure:**
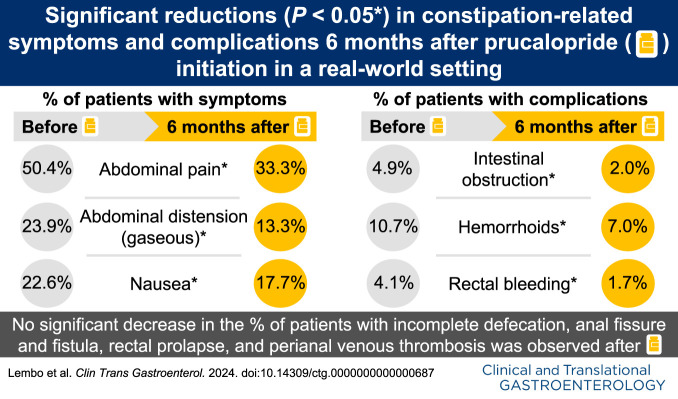

## INTRODUCTION

Chronic idiopathic constipation (CIC) is a functional bowel disorder affecting approximately 35 million adults in the United States (1). The common symptoms of CIC in adults include infrequent or incomplete bowel movements, hard stools, and straining, as well as abdominal discomfort and bloating (2). More than 70% of adults with CIC describe their symptoms as at least “somewhat bothersome” and more than half report that CIC affects their health-related quality of life (2). However, only 22%–43% of patients seek healthcare for their constipation-related symptoms (3), and prolonged duration of constipation may be associated with increased complications, including hemorrhoids, anal fissures, and rectal prolapse (4). Among patients who take medications for symptom management, more than 90% of patients rely on over-the-counter (OTC) medications (3), but are often not satisfied with the effect of OTC medications on constipation and CIC-related abdominal symptoms (5).

Prucalopride is a selective high-affinity serotonin type 4 receptor agonist that was approved in the European Union in 2009 (6) and in the United States in 2018 for the treatment of CIC in adults (7,8). The American Gastroenterological Association and the American College of Gastroenterology 2023 guidelines recommend the use of prucalopride in adults who have failed to respond to OTC medications (9). Although the efficacy and safety of prucalopride have been established in several clinical studies (10), only a few studies have reported the effect of prucalopride in a real-world setting, and these were small in size, outside of the United States, and limited to single centers (11,12). Understanding the real-world clinical challenges of CIC and treatment outcomes in a more heterogeneous patient population and setting is therefore important to facilitate informed decision-making in routine clinical practice. To this end, we aimed to evaluate the real-world impact of prucalopride treatment initiation on constipation-related symptoms and constipation-related complications in adults with CIC in the United States using US-based insurance claims data.

## METHODS

An observational, retrospective cohort analysis was performed using the latest available insurance claims data from the IBM MarketScan Commercial Claims and Encounters (MarketScan CCAE) Database and the Medicare Supplemental (MDCR) Database from January 1, 2015, to June 30, 2020. The MarketScan CCAE Database contains the combined claims data of approximately 260 self-insured employers and 40 health plans in the United States, and represents all census geographical regions, covering in total 240 million people (aged 0–64 years) insured from 1995 onward. The MDCR Database captures information on the subset of Medicare beneficiaries who possess supplemental insurance paid by their employers. The MDCR population includes active and retired employees and their Medicare-eligible dependents.

Patients eligible for this study were aged 18 years and older and had at least 1 prescription fill for prucalopride on or after April 2, 2019—the date when prucalopride became commercially available by prescription in the United States. Prescription fills for prucalopride were identified using the Generic Product Identifier and National Drug Code (see Supplementary Table 1, Supplementary Digital Content 1, http://links.lww.com/CTG/B95). The first prucalopride prescription fill date was defined as the index date. The baseline period was defined as the 6 months before the index date, and the study period was defined as 6 months after the index date (inclusive of the index date). Constipation-related *International Classification of Diseases, Tenth Revision, Clinical Modification* (*ICD-10-CM*) diagnosis codes during the 6-month baseline or study periods were used to ensure that patients treated with prucalopride had constipation. Patients were required to have 1 or more of these *ICD-10-CM* diagnosis codes: constipation, unspecified (K59.00); slow transit constipation (K59.01); outlet dysfunction constipation (K59.02); CIC (K59.04); and other constipation (K59.09). The use of additional diagnosis codes other than “CIC,” especially “unspecified constipation” and “other constipation” ensured that all patients with possible CIC were included in the study. In addition, patients were required to be enrolled in a continuous health plan for at least 6 months before the index date and at least 6 months after the index date. Patients were not followed up after the study period. Patients were excluded from the study if, during the baseline or study periods, they had 1 or more codes (*ICD-10-CM*, Healthcare Common Procedure Coding System and/or generic product identifier codes) for irritable bowel syndrome with constipation (IBS-C); drug-induced constipation or opioid supply for 45 days or more; or postoperative ileus (see Supplementary Table 2, Supplementary Digital Content 1, http://links.lww.com/CTG/B95).

The proportions of patients who had a diagnosis of constipation-related symptoms and constipation-related complications were measured during the baseline and study periods and were identified using *ICD-10-CM*, *ICD-10*-*Procedure Coding System* (*ICD-10-PCS*), or Current Procedural Terminology (CPT) diagnosis codes (see Supplementary Table 3, Supplementary Digital Content 1, http://links.lww.com/CTG/B95). The constipation-related symptoms investigated were abdominal pain, abdominal distension (gaseous), incomplete defecation, and nausea. The constipation-related complications investigated were anal fissure and fistula, intestinal obstruction, rectal prolapse, hemorrhoids, perianal venous thrombosis, perianal/perirectal abscess, and rectal bleeding.

All analyses were conducted using R version 3.6.3 and SAS version 9.4 (SAS Institute, Cary, NC). Descriptive statistics were reported; mean and SD were reported for continuous variables and frequency counts and percentages were reported for categorical variables. Statistical comparisons of constipation-related symptoms and constipation-related complications before and after prucalopride initiation were conducted using McNemar tests. Patient demographics and clinical characteristics, constipation-related symptoms, and constipation-related complications data were reported for the overall sample and separately for patients aged 18–64 years and those aged at least 65 years.

This study used deidentified Health Insurance Portability and Accountability Act–compliant data; formal consent and Institutional Review Board approval were therefore not required.

## RESULTS

**Figure 1.**
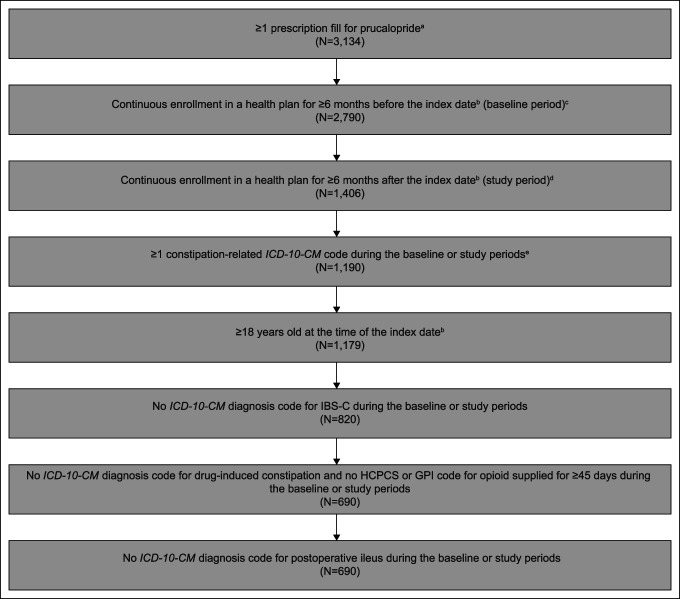
Patient disposition. ^a^All prucalopride prescription fills were on or after April 2, 2019, the date when prucalopride became commercially available by prescription in the United States. ^b^The index date was defined as the first prucalopride prescription fill date. ^c^The baseline period was defined as 6 months before the index date. ^d^The study period was defined as 6 months starting from the index date. ^e^In this study, eligible patients had ≥1 constipation-related *ICD-10-CM* diagnosis code(s). These diagnosis codes were constipation, unspecified (K59.00); slow transit constipation (K59.01); outlet dysfunction constipation (K59.02); CIC (K59.04); other constipation (K59.09). The use of additional diagnosis codes other than “CIC,” especially “unspecified constipation” and “other constipation” ensured that all patients with possible CIC were included in the study. CIC, chronic idiopathic constipation; GPI, generic product identifier; HCPCS, Healthcare Common Procedure Coding System; IBS-C, irritable bowel syndrome with constipation; *ICD-10-CM*, *International Classification of Diseases, Tenth Revision, Clinical Modification*.

This study included 690 patients. Patient disposition is shown in Figure 1. The mean (SD) age was 48.0 (14.7) years; 91.9% (n = 634) of patients were aged 18–64 years, and 8.1% (n = 56) were aged at least 65 years. Overall, 87.5% (n = 604) were women. Nearly two-thirds (63.2%; n = 436) of patients had received 1 or more constipation-related prescription medications (i.e., lubiprostone, linaclotide, or plecanatide) before the index date (Table 1). More than one-quarter of patients (26.5%; n = 183) were also receiving other constipation-related medications (i.e., lubiprostone, linaclotide, or plecanatide) in combination with prucalopride at the time of the study (Table 1).

**Table 1.** Baseline demographics and clinical characteristics of patients with chronic idiopathic constipation

Baseline demographics and clinical characteristics	Overall (N = 690)	Patients aged 18–64 yr (n = 634)	Patients aged ≥65 yr (n = 56)
Age, yr, mean (SD)	48.0 (14.7)	45.7 (12.8)	74.4 (7.1)
Sex, n (%)			
Female	604 (87.5)	563 (88.8)	41 (73.2)
Male	86 (12.5)	71 (11.2)	15 (26.8)
Charlson Comorbidity Index,^a^ mean (SD)	0.5 (1.0)	0.5 (1.0)	1.1 (1.5)
Initial dose of prucalopride, mg, mean (SD)	1.9 (0.3)	1.9 (0.3)	1.8 (0.4)
1.0 mg, n (%)	76 (11.0)	66 (10.4)	10 (17.9)
2.0 mg, n (%)	614 (89.0)	568 (89.6)	46 (82.1)
Any constipation-related comparator treatment any time before the index date,^b^ n (%)	436 (63.2)	399 (62.9)	37 (66.1)
Index drugs,^c^ n (%)			
Prucalopride monotherapy	507 (73.5)	464 (73.2)	43 (76.8)
Prucalopride combination therapy	183 (26.5)	170 (26.8)	13 (23.2)
Prucalopride and lubiprostone	26 (3.8)	24 (3.8)	2 (3.6)
Prucalopride and linaclotide	118 (17.1)	110 (17.4)	8 (14.3)
Prucalopride and plecanatide	28 (4.1)	26 (4.1)	2 (3.6)
Prucalopride and >1 other constipation-related prescription medication	11 (1.6)	10 (1.6)	1 (1.8)

a The Charlson Comorbidity Index was defined based on criteria by Charlson et al (21) and adapted by Quan et al (22).

b The index date was defined as the first prucalopride prescription fill date. Constipation-related comparator medications included lubiprostone, linaclotide, or plecanatide.

c Constipation-related prescription medications taken concurrently with the index drug included lubiprostone, linaclotide, or plecanatide.

**Figure 2.**
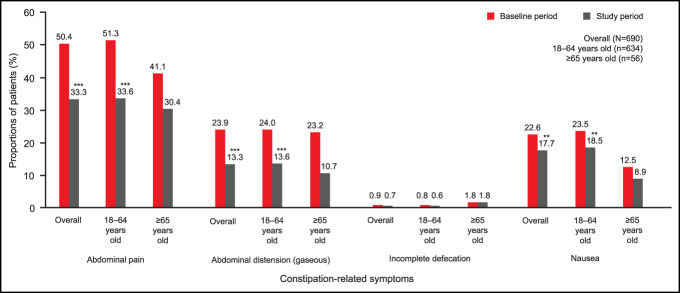
Constipation-related symptoms 6 months before prucalopride initiation (baseline period) and 6 months after prucalopride initiation (study period) in patients with CIC. *P* values for the change in constipation-related symptoms from baseline to 6 months after prucalopride initiation were calculated using McNemar tests. **P < 0.01, ***P < 0.001. CIC, chronic idiopathic constipation.

During the 6-month baseline period (i.e., before prucalopride initiation), the most commonly identified constipation-related symptom overall (based on *ICD-10-CM* diagnosis codes) was abdominal pain (50.4%, n = 348). During the 6-month study period (i.e., after prucalopride initiation), among the overall population, there were significant reductions in the proportions of patients with abdominal pain (50.4% vs 33.3%; *P* < 0.001), abdominal distension (gaseous) (23.9% vs 13.3%; *P* < 0.001), and nausea (22.6% vs 17.7%; *P* < 0.01), compared with the baseline period. The reductions from baseline in the proportion of patients aged 18–64 years with these symptoms compared with the study period were also significant (abdominal pain, 51.3% vs 33.6%; *P* < 0.001; abdominal distension [gaseous], 24.0% vs 13.6%; *P* < 0.001; and nausea, 23.5% vs 18.5%; *P* < 0.01). However, among patients aged at least 65 years, there were numerical but not statistically significant reductions in the proportion of patients with these symptoms during the 6-month study period. The occurrence of incomplete defecation (as reported by *ICD-10-CM* diagnosis codes) was low during the 6-month baseline period, and incomplete defecation was not significantly reduced during the 6-month study period in the overall population (0.9% vs 0.7%; *P* = 1.0) or among patients aged 18–64 years (0.8% vs 0.6%; *P* = 0.1). Among patients aged at least 65 years, the occurrence of incomplete defecation was unchanged (Figure 2).

**Figure 3.**
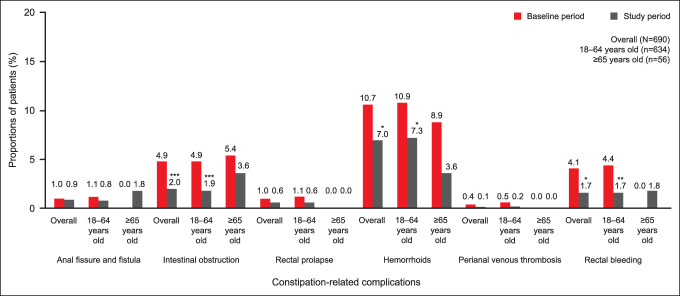
Constipation-related complications 6 months before prucalopride initiation (baseline period) and 6 months after prucalopride initiation (study period) in patients with CIC. All constipation-related complications for which at least 1 occurrence was recorded in all patients; no occurrence of perianal/perirectal abscess was recorded. *P* values for the change in constipation-related complications from baseline to 6 months after prucalopride initiation were calculated using McNemar tests. *P < 0.05, **P < 0.01, ***P < 0.001. CIC, chronic idiopathic constipation.

Constipation-related complications (based on *ICD-10-CM*, *ICD-10-PCS*, or CPT diagnosis codes) occurred in only a small proportion of patients overall, both before and after prucalopride initiation. Compared with the 6-month baseline period, there was a significant reduction during the 6-month study period in the proportions of patients overall with hemorrhoids (10.7% vs 7.0%; *P* < 0.05), intestinal obstruction (4.9% vs 2.0%; *P* < 0.001), and rectal bleeding (4.1% vs 1.7%; *P* < 0.05) (Figure 3). Among patients aged 18–64 years, there were significant reductions during the 6-month study period in the proportions of patients with these constipation-related complications: hemorrhoids (10.9% vs 7.3%; *P* < 0.05); intestinal obstruction (4.9% vs 1.9%; *P* < 0.001); and rectal bleeding (4.4% vs 1.7%; *P* < 0.01) (Figure 3). Changes in the proportions of patients aged at least 65 years with constipation-related complications were not significantly different 6 months after the initiation of prucalopride (Figure 3).

## DISCUSSION

This real-world, observational, retrospective cohort study found that, based on *ICD-10-CM*, *ICD-10-PCS*, and CPT diagnosis codes, treatment with prucalopride was associated with statistically significant reductions in a number of constipation-related symptoms (abdominal pain, abdominal distension [gaseous], and nausea) and constipation-related complications (hemorrhoids, intestinal obstruction, and rectal bleeding) over 6 months in the overall population of adults with CIC. When analyzed by age subgroup, there were statistically significant reductions in these constipation-related symptoms and constipation-related complications among patients aged 18–64 years. However, among patients aged at least 65 years, which comprised a relatively small proportion of the total study population (8.1%, n = 56/690), prucalopride was associated with numerical but not statistically significant reductions in constipation-related symptoms and complications.

The efficacy and safety of prucalopride in patients with CIC have been demonstrated in an analysis that combined data from 6 double-blind randomized controlled studies (10). Longer-term clinical studies (lasting ≥6 months) of prucalopride have also been conducted: Pooled data of patients from 3 double-blind, phase 3 randomized controlled studies showed that the improvement in patient satisfaction with bowel function observed after 12 weeks of prucalopride treatment, as determined by the average Patient Assessment Constipation-Quality of Life questionnaire, was maintained for up to 18 months during 2 open-label extension studies (13). By contrast, a long-term (24-week) randomized controlled study of prucalopride demonstrated that there was no significant difference between the proportions of patients who achieved the primary endpoint, 3 or more spontaneous complete bowel movements per week, in the prucalopride 2 mg and placebo treatment arms (14). This inconsistency highlights the need for evidence of the effectiveness of prucalopride from real-world studies.

In this real-world study of prucalopride in patients with CIC, it was notable that more than 50% of patients had a diagnosis code of abdominal pain, typically the hallmark of IBS (15), during the baseline period. Although abdominal pain may be present in patients with CIC, the Rome IV criteria state that it should not be the predominant symptom in CIC, which is distinguished from IBS-C by the lack of recurrent abdominal pain (i.e., at least once a week over a period of 3 months) (16). However, it is worth emphasizing that the clinical presentations of CIC and IBS-C exist on a spectrum (16). Accordingly, in a real-world setting, differentiating between these disorders, and therefore assigning a patient the appropriate diagnosis code, can be difficult (17). Therefore, despite the exclusion of patients from this study with a diagnosis code for IBS-C, it is possible that some patients may have had IBS-C rather than CIC. It should also be noted that other more general errors and inaccuracies with assigning diagnosis codes have been reported (18), which may include erroneous or intentional miscoding for a nonapproved indication. Likewise, it is also relevant to acknowledge that more than 60% of patients in this study had previously used constipation-related prescription medications before starting treatment with prucalopride. As a result, this study may be representative of patients for whom previous treatment did not improve symptoms, suggestive of a more refractory patient population.

This study is limited by several factors including the lack of generalizability of results from commercially insured patients to other patient populations in the United States, such as Medicaid beneficiaries or the uninsured, as well as other populations globally. Furthermore, the limited proportion of patients aged at least 65 years (8.1%) may have made observing trends in this population more difficult. In addition, the 6-month study period did not allow for the assessment of any potential long-term benefits associated with prucalopride treatment. Although the primary inclusion criterion of this study was that patients had at least 1 prescription fill for prucalopride, which is only indicated for the treatment of adults for CIC, this study included patients who had constipation-related *ICD-10-CM* diagnosis codes other than “CIC” (e.g., “constipation, unspecified” and “other constipation”). This ensured that all patients with possible CIC were included, given that other closely related diagnosis codes are often used to describe CIC in clinical practice. Likewise, as discussed above, although patients who had an *ICD-10-CM* diagnosis code for IBS-C were excluded, this study may have included some patients whose clinical presentation typically better corresponds to that seen in IBS-C than CIC owing to the difficulty of discriminating between the 2 indications in a real-world setting. Nevertheless, the eligibility criteria for this study were chosen with the intention of including all patients with possible CIC, given that clinical case data were not available in the claims data used. Moreover, some diagnoses may not have been assigned to any of the specific constipation-related symptoms examined, leading to coding inaccuracies and the underreporting of constipation symptoms. However, this is unlikely to have affected the direct comparison of the baseline and study period data, given that there was an observed decrease in both constipation codes and constipation symptom codes during the study period relative to the baseline period (data not shown). It should be noted that the symptoms that were selected for investigation in this study are among some of the most commonly observed in patients with CIC (2,16); less common symptoms of CIC were not examined as part of this study. In addition, symptoms were determined through *ICD-10-CM*, *ICD-10-PCS*, and CPT diagnosis codes, which do not fully measure symptoms or disease severity, but rather facilitate administrative claims made for reimbursement purposes. This in turn may have resulted in an underreporting of constipation-related symptoms and complications. Furthermore, constipation-related complications could have been caused by other conditions and may not have been attributable solely to constipation. However, these constipation-related complications were selected for investigation because they are some of the most frequently observed concurrent conditions in patients with chronic constipation (19,20). It is also possible that patients may not have used prucalopride as prescribed despite having a prescription fill, which could have affected the study findings.

In summary, this large, observational, real-world, retrospective claims study found that treatment with prucalopride was associated with significant improvements in clinician-coded constipation-related symptoms and constipation-related complications in adults with CIC after 6 months. More real-world, longer-term studies of prucalopride are needed, especially in patients aged at least 65 years.

## CONFLICTS OF INTEREST

**Guarantor of the article:** Anthony Lembo, MD, FACG.

**Specific author contributions:** M.L., B.T., and M.B.: study conception. E.T., S.D., R.A., B.W., and S.P.: data analysis. All authors contributed to data interpretation. All authors contributed to the drafting of the manuscript and revising it for important intellectual content. All authors reviewed and approved the final draft of the manuscript for submission.

**Financial support:** This study was funded by Takeda Pharmaceuticals USA, Inc.

**Potential competing interests:** A.L. has received consultancy fees from AEON BioPharma, Alkermes, Allakos, Anji Pharmaceuticals, Ardelyx, Arena Pharmaceuticals, Atmo Biosciences, Biomerica, Gemelli Biotech, Ironwood Pharmaceuticals, Neurogastrx, OrphoMed, Pfizer, QOL Medical, Shire (a Takeda company), Takeda Pharmaceuticals, and Vibrant Pharma; has received advisory board fees from Evoke Pharma; and is a stockholder of Allurion, Bristol Myers Squibb, and Johnson & Johnson. B.D.C. has received consultancy and speaker fees from AbbVie, Alnylam Pharmaceuticals, Ardelyx, Arena Pharmaceuticals, QOL Medical, Salix Pharmaceuticals, and Takeda Pharmaceuticals. R.A. is an employee of Analysis Group Inc., and received funding from Takeda Pharmaceuticals USA, Inc. to perform this study. B.T. and M.L. are employees of Takeda Pharmaceuticals USA, Inc. and stockholders of Takeda Pharmaceutical Company Limited. M.B. is an employee of Ironwood Pharmaceuticals, Inc. but was an employee of Takeda Pharmaceuticals USA, Inc. and a stockholder of Takeda Pharmaceutical Company Limited at the time this study was conducted. P.F. has received consultancy and speaker fees from Ferring Pharmaceuticals/Rebiotix, Merck ' Co., Seres Therapeutics, and Takeda Pharmaceuticals; and has received advisory board fees from Ferring Pharmaceuticals/Rebiotix, Probiotec Ltd., Sanofi, SERES Therapeutics, and Takeda Pharmaceuticals. B.M. has received consultancy and speaker fees from Alnylam Pharmaceuticals; has received research grant support from Atmo Biosciences, ReStalsis Health, Salix Pharmaceuticals/Bausch Health, and Takeda Pharmaceuticals; has received advisory board fees from AbbVie, Allergan, Alnylam Pharmaceuticals, Biora Therapeutics (formerly Progenity), Coloplast, Medtronic, Salix Pharmaceuticals/Bausch Health, and Takeda Pharmaceuticals; and is an inventor with 2 patents from Atrium Health and the University of Miami. At the time of this study, B.W., E.T., S.D., and S.P. were employees of Analysis Group, Inc. and received funding from Takeda Pharmaceuticals USA, Inc. for conducting this study.

**Study Highlights**

**WHAT IS KNOWN**

✓ Chronic idiopathic constipation is a functional bowel disorder affecting approximately 35 million adults in the United States.
✓ Prucalopride is a serotonin type 4 receptor agonist, approved in the European Union in 2009 and in the United States in 2018 for adults with chronic idiopathic constipation.
✓ The efficacy and safety of prucalopride have been demonstrated in clinical studies; however, real-world data are limited.

**WHAT IS NEW HERE**

✓ Using US-based insurance claims data, abdominal pain and hemorrhoids were the most commonly reported constipation-related symptom and complication, respectively.
✓ Prucalopride was associated with reductions in constipation-related symptoms and constipation-related complications in a real-world setting.

## Supplementary Material
