# Supplementary material for: Clinical Outcomes Before and After Prucalopride Treatment: An Observational Study in Patients With Chronic Idiopathic Constipation in the United States
Source: Clin Transl Gastroenterol. 2024 Feb 15;15(5):e00687. doi: 10.14309/ctg.0000000000000687 (PMC11124638; doi:10.14309/ctg.0000000000000687)
Supplement: Supplementary file 1 [file ct9-15-e00687-s001.docx]

**Supplemental Information**

**Supplemental Table 1.** NDC and GPI codes to identify the prescription fills for prucalopride.

| Treatment | NDC code | GPI code |
| --- | --- | --- |
| Prokinetics | **–** | 5230x 525550x  5256x 50100020x |
| Prucalopride | 54092054601 54092054701 54092054702 54092054703 | **–** |

GPI, Generic Product Identifier; NDC, National Drug Code.

**Supplemental Table 2.** ICD-10-CM, HCPCS and GPI codes for the exclusion criteria.

| **Diagnosis** | **ICD-10-CM code** | **HCPCS code** | **GPI code** |
| --- | --- | --- | --- |
| Irritable bowel syndrome with constipation  Mixed irritable bowel syndrome  Irritable bowel syndrome without diarrhea  Drug-induced constipation  Opioid (≥45-day supply)  Postprocedural intestinal obstruction | K58.1  K58.2  K58.9  K59.03  –  K91.3x | –  –  –  –  J0745, J2270, J2271, J2275, S0093, J3010, J1170, S0092, J1960, J2175, J2180, J1230, S0109, J2410, J0592, J0595, S0009, S0012, J2300, J3070, J0570, J0571, J0572, J0573, J0574, J0575, Q9991, Q9992, Q0167, Q0168  – | –  –  –  –  65x  – |

ICD-10-CM, International Classification of Diseases. Tenth Revision, Clinical Modification; HCPCS, Healthcare Common Procedure Coding System; GPI, Generic Product Identifier.

**Supplemental Table 3.** ICD-10-CM, ICD-10-PCS and CPT codes for constipation-related symptoms and constipation-related complications.

| **Diagnosis** | **ICD-10-CM code** | **ICD-10-PCS code** | **CPT code** |
| --- | --- | --- | --- |
| **Constipation-related symptoms**  Abdominal pain  Abdominal distension (gaseous)  incomplete defecation  Nausea  **Constipation-related complications**  Anal fissure and fistula  Intestinal obstruction  Rectal prolapse  Hemorrhoids  Perianal venous thrombosis  Peri-anal/peri-rectal abscess  Rectal bleeding | R10.0, R10.1x, R10.2, R10.3x, R10.8x, R10.9  R14.0  R15.0  R11.0, R11.2  K60.0, K60.1, K60.2, K60.3, K60.5  K56.x  K62.3  K64.0, K64.1, K64.2, K64.3, K64.8, K64.9  K64.5  K61.0, K61.1, K61.2  K62.5 | –  –  –  –  0D8R0ZZ, 0D8R3ZZ, 0DBQ0ZZ, 0DBQ3ZZ, 0DBQ4ZZ, 0DQQ0ZZ, 0DQQ3ZZ, 0DQQ4ZZ, 0DQQ7ZZ, 0DQQ8ZZ, 0DQQXZZ, 0H89XZZ  3E1H78Z, 0DF80ZZ ‑ 0DF8XZZ,  0DFA0ZZ ‑ 0DFAXZZ, 0DFB0ZZ ‑ 0DFBXZZ, 0DFE0ZZ ‑ 0DFEXZZ, 0DFF0ZZ ‑ 0DFFXZZ, 0DFG0ZZ ‑ 0DFGXZZ, 0DFH0ZZ ‑ 0DFHXZZ, 0DFK0ZZ ‑ 0DFKXZZ, 0DFL0ZZ ‑ 0DFLXZZ, 0DFM0ZZ ‑ 0DFMXZZ, 0DFN0ZZ ‑ 0DFNXZZ, 0DFP0ZZ ‑ 0DFPXZZ, 0DFQ0ZZ ‑ 0DFQXZZ, 0DNP0ZZ, 0DNP3ZZ, 0DNP4ZZ, 0DNP7ZZ, 0DNP8ZZ, 0WFP0ZZ ‑ 0WFPXZZ  0DSPXZZ, 0DSP0ZZ, 0DSP4ZZ, 0DSP7ZZ, 0DSP8ZZ, 0DUP0JZ, 0DUP4JZ  065Y0ZC, 065Y3ZC, 065Y4ZC, 06BY0ZC, 06BY3ZC, 06BY4ZC, 06LY0CC, 06LY0DC, 06LY0ZC, 06LY3CC, 06LY3DC, 06LY3ZC, 06LY4CC, 06LY4DC, 06LY4ZC  –  0D9R00Z, 0D9R30Z, 0D9R40Z, 0D9Q00Z, 0D9Q0ZZ, 0D9Q30Z, 0D9Q3ZZ, 0D9Q40Z, 0D9Q4ZZ, 0D9Q70Z, 0D9Q7ZZ, 0D9Q80Z, 0D9Q8ZZ, 0D9QX0Z, 0D9QXZZ, 0D9Q40Z, 0D9Q4ZZ  – | –  –  –  –  46020, 46030, 46060, 46200, 46257, 46258, 46261, 46262, 46270, 46275, 46280, 46285, 46288, 46505, 46706, 46707, 46715, 46716, 46940, 46942, 0170T  45915, 91123, 99511, 45321, 45337, 45393, 74283, 44055, 44050, 44363, 44390, 45307, 45332, 45379, 46608, 44615, 45150, 46700, 46705  45130, 45135, 45400, 45402, 45505, 45520, 45540, 45541, 45550, 45900, 46750, 46753, 46754  45350, 45398, 46221, 46250, 46255, 46257, 46258, 46260, 46261, 46262, 46500, 46930, 46934, 46935, 46936, 46945, 46946, 46947, 46948, 0249T  46083, 46320  46040, 46045, 46050  – |

CPT, Current Procedural Terminology; ICD-10-CM, International Classification of Diseases. Tenth Revision, Clinical Modification; ICD-10-PCS, International Classification of Diseases. Tenth Revision, Procedure Coding System.
